# Drug use prevention projects in schools in Vitória, Brazil: quality analysis and improvement proposals

**DOI:** 10.1186/s41155-016-0056-2

**Published:** 2017-01-11

**Authors:** Elizeu Borloti, Maria Victoria Hidalgo García, Virginia Sanchez Jiménez, Maria Fátima Oliver Sudbrack

**Affiliations:** 1 Universidade Federal do Espírito Santo – UFES, Av. Fernando Ferrari, 514 - Goiabeiras, Vitória, ES CEP 29.075-910 Brazil; 2 Universidad de Sevilla – US, C/ S. Fernando, 4, C.P. 41004 Sevilla, Spain; 3 Universidade de Brasília – UnB, SCS Quadra 4, Ed. Anápolis, 40 andar, Brasília, DF CEP 70300-000 Brazil

**Keywords:** Drug use prevention, School programs, Teacher training

## Abstract

Adolescents living in vulnerable regions are more exposed to risk factors for drug use. The prevention of such use in school is a public policy that needs evaluation. Based on technical criteria and derived from a mixed research, this article analyses the quality of school-based prevention of drug use in Vitória, state of Espírito Santo, Brazil, and proposes improvements. A checklist of quality elements was completed with data from 16 projects proposed by 99 teachers from public schools. In 10 projects (62.5%), the approximate quality index was above 0.50. The majority of projects fulfilled the requirement of theoretical foundation (81.25%) and some of the methodological (93.75%), design (75%) and implementation (62%) requirements. Other requirements were absent: the majority were not designed by the whole school community (87.5%), and the participation of the family (62.5%) or the students as mediators (62.5%) was not considered. In general, contents of life skills (87.5%), positive relationships and alternative activities to drug use (56.25%) were not included. Activities for reinforcing the content were not described in any of the projects, and evaluation activities were described in only a few (31.25%). Many projects did not describe the inclusion of the project in the school curriculum (62.5%). Although, considering all items of effectiveness, regardless of their weight, more than half of the projects had an above average quality. The present items provide quality to the projects, whereas absent items indicate shortcomings to be improved using some of the measures described in this study.

## Background

Drug abuse is a serious public health problem related to morbidity and mortality statistics (Griffin and Botvin 2010). Adolescents living in vulnerable regions are exposed more and earlier (De Vincenzi and Bareilles 2011; Giacomozzi et al. 2012) to risk factors for drug use (Fabrizio et al. 2013; MacLean et al. 2013). Current data show that drug use before 10 years old occurs in approximately 5% of Brazilian students. While the frequency of this use is lower in public schools in Brazil than in private schools, heavy or daily drug use is more frequent among public school students of low socioeconomic levels (Carline et al. 2010).

Although the school is usually a protective context for development, the social relationships between students can act as a risk factor for drug use of some students by others, especially concerning the use of alcohol among boys (Kwan et al. 2015). In addition, there are risk factors of the school per se, listed in the classic article by Gottfredson (1987), such as low-quality education. A recent Thai study (Wongtongkam et al. 2014) showed that little involvement with the school is a strong risk factor, increasing the chances of using *club drugs* 10-fold and tripling the chances of using solvents. These and other factors are more incisive in the adolescent’s transition to secondary school, a level that predisposes students to experimentation (Piko and Kovács 2010).

In secondary or elementary school, drug use prevention is important (Becoña 2002) because it aims to (a) delay the age of onset of use; (b) limit the number and type of substances used; (c) prevent the progress of use to abuse or dependency; (d) reduce use harms; (e) teach rules for responsible use (as it is part of the culture); (f) create contingencies that strengthen protective factors and weaken risk factors; and (g) create cultural and community contingencies that favour the acquisition and maintenance of a healthy lifestyle. Currently, such objectives have been advocated by several researchers (e.g. Cerkez et al. 2015; Hollen and Ortiz 2015) and by government agencies, such as the European Monitoring Centre for Drugs and Drug Addiction (EMCDDA 2011) and the National Secretariat for Drug Policy (Secretaria Nacional de Políticas sobre Drogas (SENAD)) of the Brazilian government (Sudbrack et al. 2014).

The advocacy of such objectives is justified because the risk behaviours of adolescents impact the health and economy of a country (Reyna and Farley 2006). Therefore, Brazil has drug use prevention as one of the axes of the *Integrated Plan to Combat Crack and other Drugs* (Decree no. 7179 of May 20, 2010). Some of the global actions of this plan for prevention in the community context are well known, such as those restricting the age for purchasing alcohol and tobacco (Reyna and Farley 2006). Others have been taught in the “Drug Use Prevention Course for Educators of Brazilian Public Schools”, a partnership established since 2004 between the University of Brasilia, the Ministry of Education and SENAD. The goal of that course is to train educators in designing prevention projects in schools, a privileged place for (a) resources that can be used for prevention; (b) well-trained teachers to implement prevention strategies; (c) a schedule that allows the inclusion of preventive activities; (d) access to the entire child and adolescent population; (e) positive influences on different behaviours, in addition to academic ones; and (f) the capacity to encourage prevention with the participation of students and families (Ramos et al. 2010).

These advantages of the school for prevention are many, but not so many as the practices that have been developed in Spanish (Espada et al. 2015) or American (Jackson et al. 2012) prevention programs in schools. While this variability is positive from one point of view, it also prevents a preview of what exactly determines the effectiveness of school-based prevention. Thus, since the 1980s, effectiveness has been the focus of research. However, it was especially from the 2000s that meta-analyses and systematic reviews of those research studies grouped its conclusions and indicated what should be the elements of this effectiveness. The same period in which the effectiveness of some specific preventive strategies was investigated.

Fernández et al. (2002) identified, in a review of five meta-analyses published between 1990 and 1999, that even programs with potential produce mild effects and that the more effective programs were those (a) that adopted a social influence model; (b) with interactive methodology; (c) that were applied by professors and students; and (d) that were thoroughly implemented and evaluated. Concomitantly, Cuijpers (2002), after analysing three meta-analytical studies, six interventional studies with manipulation of mediating variables and four comparative studies of programs with and without specific elements indicated seven quality criteria for school-based prevention: (a) outcome measure prediction; (b) interactive methodology; (c) adoption of the social influence model; (d) reinforcement of rules of commitment and intention not to use drugs; (e) involvement of the community; and (f) of students as mediators; and (g) life skills content.

Six years later, Faggiano et al. (2008), in a review that categorized 29 studies according to their focus on skills, affect or knowledge, concluded that affective (e.g. those for improving self-esteem) and knowledge programs improve decision-making and/or “knowledge on” drugs, but both do not exceed the effectiveness of skills programs. Then, Peters et al. (2009) also reported this effectiveness in a review of reviews. They described that strong evidence that effectiveness is dependent on five elements: (a) the underlying theory; (b) the social influence model; (c) skills training; (d) training of facilitator students; and (e) multidimensional objectives. However, in his systematic review, Midford (2010) appointed the social influence model based on the social learning theory as limited for drug prevention.

More recently, the skills training was also evident as effective for drug use prevention in the 18 heterogeneous experimental studies reviewed by Jackson et al. (2012). Most interventions described on such experimental studies have produced some expected result and were more effective when they simultaneously focused on the skills required in various risk and protection domains. Those skills are generically called life skills, already recognized as the “primary focus” of school-based prevention (Winters et al. 2007, p. 375). This primacy was reported in a systematic review of 17 recent studies, published by Jiménez et al. (2014), that identified 29 elements associated with the effectiveness of prevention at school, highlighting the training of life skills.

Mild positive effects of school-based prevention on alcohol use were observed in the meta-analysis conducted by Strøm et al. (2014) with a sample of 28 articles published between 1999 and 2014. Espada et al. (2015) also found low effectiveness in 21 studies on the effectiveness of Spanish programs published between 2001 and 2011, while the more effective studies were those (a) with the aim of changing attitudes; (b) based on health education or social learning theory; (c) that used a combination of oral, written and audio-visual support material; and (d) that were implemented by teachers and other professionals. In relation to the social learning theory as an underlying prevention model, the systematic review of periodical literature by Jiménez et al. (2014) indicated it as an effective element. The cognitive-behavioural (Sandler et al. 2014)—including the social learning theory—and the health education models were most related to effectiveness (Espada et al. 2015).

While meta-analyses and systematic reviews were conducted, some studies stand out for investigating the effectiveness of specific preventive strategies. The game “Challenges” taught problem-solving and modified the beliefs that drugs can solve conflicts (Araujo et al. 2011). Team sports, compared to technical or strength activities, are usually associated with greater involvement with alcohol and lower involvement with tobacco and marijuana (Wichstrom and Wichstrom 2009); in particular, boys’ participation in individual sports is a protective factor for alcohol use (Cerkez et al. 2015). A social network is effective in prevention though this effectiveness depends on the group processes inherent to it (social network-tailored prevention was effective and reduced the likelihood of drug use only in a social network that reinforced non-use; Valente et al. 2007).

In addition to differences in details, Jiménez et al. (2014) noted a lack of rigor in designs for the evaluation of prevention in the studies reviewed. Moreover, there were conflicting results on the effectiveness elements described in the literature, such as the involvement or not of students as facilitators. Gottfredson and Wilson (2003) also questioned the meaning of “intensity” of the intervention. According to Arco and Fernández (2002), variations in method and in the evaluation may explain these contradictions, making the analysis of the essential elements of school-based drug use prevention programs relevant to minimize dissensions. In addition, there are other controversies in this field of study, including the disclosure of only “what worked”, as reported by Holder (2008). The conclusion of Suárez et al. (2014) is related to this finding: professional experts from four schools of Seville, Spain, considered that the preventive actions they develop are effective, while students had an opposite opinion.

In short, the results available on quality elements of school-based drug use prevention programs are still disaggregated, and some are not consensual (Jiménez et al. 2014); more rigorous evaluations of interventions need to be made and implemented in public policies (Espada et al. 2015). Table 1 summarizes the effectiveness elements of drug use prevention planning at school, with definitions and support references (cited or not in the review or meta-analytic studies referred in the preceding paragraphs), ranking considering the strength-of-evidence in the essential elements of effectiveness (also indicated as “*I*”, on Table 2). Each reference on the right column clearly cites the element on the left column as element of effectiveness of school-based prevention of drug use. Ramos et al. (2010) and Jiménez et al. (2014) cited most of them as key of effectiveness. According to Jiménez et al., the elements were ranked by the strength-of-evidence in their effectiveness, indicating the weight of the evidence observed for each of them. The authors took into account the number of articles that evaluated each element and the percentage of them that demonstrated or not the effectiveness of each of them to deduct a score with a weight ranged from 0 to 1 points.

**Table 1:** Effectiveness elements of drug use prevention planning at school

Element	Definition	Reference
Life skills content	Training in personal skills such as negotiation, self-esteem promotion and decision-making strategies	Winters et al. (2007): It is the “primary focus” (p. 375) of the adolescent-targeted prevention.
Participation of out-of-school agents	Active participation of the community in the project implementation, through partnership with companies and health and security agents, etc.	Sandler et al. (2014): Programs with school-community partnership show “slightly higher” effects (p. 253) when compared to those without this partnership.
Participation of students as mediators	Students play an active role in the implementation of activities.	Fernandez et al. (2002): The most effective interventions are implemented with the participation of the students.
Positive social relationships content	Activities that promote the creation or strengthening of positive social networks (e.g. family-student, family-school, student-community).	Markham et al. (2012): A positive school ethos makes drug use by students less likely.
Interactive/experiential methodologies	Techniques that nurture contact and communication between participants; practice of taught skills or the experience of their own experiences and reflections, in a constructive and reflective way.	Gázquez et al. (2009): The most effective prevention methods are the interactive ones.
Participation of families	Family is actively involved in project implementation, at least in some of the proposed activities	Peters et al. (2009): Parental involvement is an additional preventive element, reducing the likelihood of using drugs and of inadequate sexual and eating behaviours.
Quality evaluation	Rigorous and of quality evaluation	Winters et al. (2007): The evaluation of needs produces a realistic plan; the evaluation of the outcomes of this plan shows the progress and the effectiveness of the prevention plan.
Content reinforcement	Sessions to be held as reinforcement after the intervention ends	Gázquez et al. (2009): Preventive effects typically do not last or gradually decrease, suggesting the need for reinforcement sessions.
Design based on the participants’ needs	Content and methodology adjusted to the particularities or needs of the participants, including age, gender, culture and/or socioeconomic status	Winters et al. (2007): “Effective programs appreciated the importance that prevention has to be adjusted to maximize their relevance for the target populations” (p. 375).
Adoption of a theoretical model	Explanation of the theory underlying the project	Fernandez et al. (2002): The most effective interventions address the social influence on drug use.
		Sandler et al. (2014): Programs following the cognitive behavioural model show broader preventive effects.
Duration/periodicity	Intensity of the intervention	Cuijpers (2002): More intense programs are more effective than less intense ones.
		Gottfredson and Wilson (2003): It is important to pay attention to a more sensitive measure of the intensity of interventions because the longest ones are not necessarily the most effective.
		Gázquez et al. (2009): Preventive effects typically do not last or gradually decrease, suggesting the need for ongoing interventions.
Design by the whole school community	Participation of the whole school community in the project	Rowling (2003): “The participation of all school community members is an essential principle that underpins health promotion practice” (p. 17).
Inclusion in the school curriculum	Project is part of the school curriculum and of the educational objectives of the school.	Berkowitz and Bier (2005): The integration of prevention of risk behaviour in the academic curriculum is a strategy of effective programs.
Alternative activities to drug use	Extracurricular activities, generally of positive social engagement, alternative to those with health risk	Carmona and Stewart (1997): More effective programs include alternative activities; preferably, these activities should be part of the everyday network of community resources.

**Table 2:** Checklist of project quality requirements with strength-of-evidence of effectiveness of the essential effectiveness elements (*I*)

Quality requirement		*I*
Theoretical foundation	Theoretical model	0.24
Objectives	Clear definition	–
	Focus on primary prevention	–
	Focus on secondary prevention	–
	Focus on tertiary prevention	–
Participants	Families	0.29
	Out-of-school community	–
	Elementary school students	–
	Secondary school students	–
Content	Life skills	0.53
	Knowledge about drugs	–
	Positive social relationships	0.29
	Alternative activities to drug use	0.06
Methodology	Interactive/experiential	0.29/0.18
	Informative	0.24
	Written printed material	–
	Audio-visual/internet material	–
Characteristics	Bimonthly duration	0.21
	Semi-annual duration	
	Annual duration	
	Daily periodicity	
	Weekly periodicity	
	Monthly periodicity	
	Semi-annual periodicity	
	Implementation by agents with specific training	0.29
	Implementation by out-of-school agents	0.18
	Implementation by students as mediators	0.35
	Design by the whole school community	0.18
	Design based on the participants’ needs	0.24
	Inclusion in the school curriculum	0.06
	Content reinforcement	0.24
Evaluation	Pre- and post-test	0.28
	Process evaluation	
	Control group	
	Qualitative evaluation	

The only article available on prevention programs in Brazilian schools analysed 133 articles published from 1999 to 2001 (Canoletti and Soares 2005) and showed that the goal of these early programs was generally to inform about drugs, with many of the elements in Table 1 being absent. Therefore, it becomes relevant to assess the quality of the drug use prevention programs designed by educators and applied in Brazilian schools. The “Prevention Course” of the current Brazilian public policy for continuing education was in force in the decade after the one evaluated by Canoletti and Soares (2005), supported by updated knowledge (Sudbrack et al. 2014) that, for example, interactive methods (Gázquez et al. 2009) and the participation of the whole community (Sandler et al. 2014) are more related to effectiveness. However, the quality of these projects has yet to be comparatively analysed for adjusting this policy (experience and research in 10 years of training of public school teachers for the prevention of drug use were reported by Sudbrack et al. 2015). Thus justified, and in light of existing data on characteristics that determine the effectiveness of prevention programs, this study has two aims: (1) to analyse the quality of school-based drug use prevention projects in Vitória, state of Espírito Santo (ES), Brazil, and (2) to propose improvements in these projects.

## Method

This mixed study evaluated 16 drug use prevention projects in public schools in Vitória, ES, Brazil. Quality elements were identified from data source, and its presence or absence was quantified. This data source was generated by 99 teachers who attended the fifth edition (2012–2013) of the national training program, “Drug Use Prevention Course for Educators of Brazilian Public Schools”. From 2004 to 2014, this program has already trained more than 100,000 teachers, allowing them to access the content in interactive online devices through a Moodle Platform. The 99 authors of the projects are part of the 71,856 educators from 9202 schools selected for the fifth edition of the course. This edition was selected due to its differences from the previous editions, namely, greater absolute number of teachers trained (31,448) and longer training period (eight months, previously four; and 180 h, previously 120).

The choice of Vitória, ES, also has its justification: along with Lauro de Freitas (state of Bahia (BA)) and Contagem (state of Minas Gerais (MG)), Vitória, is part of the United Nations Office on Drugs and Crime (UNODC 2013) program “Security with Citizenship: preventing violence and strengthening citizenship with a focus on children, adolescents and youths in vulnerable conditions in Brazilian communities”. Particularly, because of the vulnerability in of the region of Greater São Pedro, Vitória, was elected among the 82 metropolitan regions of Brazil applying for the program’s resource.

### Instruments

1. Project fact sheet. As in the study by Ramos et al. (2010), the following information was recorded: (a) project identifier (number and keyword); (b) title; (c) school contact; (d) authorship; (e) application territory; (f) application context; (g) target population; (h) content of the intervention; and (e) presentation format.
2. Checklist of project quality elements. The quality elements were developed based on the studies by Jiménez et al. (2014) and Espada et al. (2015). Conclusions regarding the quality of programs were adapted for the analysis of project quality using an Excel spreadsheet that recorded the following elements: (a) theoretical foundation (theoretical model); (b) objectives (clear definition, focus on primary, secondary and tertiary prevention); (c) participants (families, out-of-school community, elementary and secondary school students); (d) content (life skills, knowledge about drugs, positive relationships, alternative activities to drug use); (e) methodology (interactive/experiential, informative, written printed material, audio-visual/internet material); (f) characteristics (bimonthly, semi-annual and annual durations; daily, weekly, monthly and semi-annual periodicities; school hours, out-of-school hours; implementation by agents with specific training, implementation by out-of-school agents, implementation by students as mediators; design by the whole school community, design based on the participants’ needs; inclusion in the school curriculum; content reinforcement); and (g) evaluation (pre- and post-test, process evaluation, control group, qualitative evaluation). Table 2 reproduces the instrument’s content. Elements directly associated with effectiveness are indicated with *I*, the strength-of-evidence of effectiveness. According to the systematic review of periodical literature conducted by Jiménez et al., *I* indicates the number of articles that evaluated each element and the percentage of them that demonstrated or not its effectiveness. The categories are not mutually exclusive (e.g. some activities of project 1 have monthly periodicities, while others occur weekly).
3. Sheet of improvement proposals. As described by Ramos et al. (2010) and based on the data from instrument 2, the following were stated: (a) the shortcomings of the project; (b) the proposal for improving the project; and (c) the justifications and concrete measures.

### Data processing and analysis

Three readings of each project were conducted for simultaneously filling instruments 1 and 2.

After reading the 16 projects, data from the checklist were analysed according to the total presence (Y = yes) or absence (N = no) of the elements related to the strength-of-evidence of effectiveness (Jiménez et al. 2014), according to the content of each project. This analysis enabled calculating the *approximate project quality index*, considering as good-quality projects those that exceeded the 0.50 index (the cut-off index used in the study by Ramos et al. 2010). The checklist inserted into an Excel spreadsheet was scored using descriptive statistics, considering the percentage of elements of effectiveness present in the project (within the total of 19 elements in Table 2, considering one and any intensity or “evaluation”). Duration and frequency were considered as elements of the intervention intensity; any form of evaluation was considered; the content “knowledge about drugs” has an *I* of 0.24 and was considered as an “informative” methodology.

The analysis of the absence of effectiveness elements indicated the proposals for improving the quality of projects with an index below 0.50. As suggested by Ramos et al. (2010), the proposals focused the elements with more weight of the evidence of effectiveness (“*I*” column on Table 2) and less present in the projects. The sheets of improvement proposals indicated the deficiencies observed in the previous quantitative evaluation of effectiveness elements. All absent elements with high strength-of-evidence of effectiveness in most projects indicated deficiency.

## Results

Table 3 shows the presence or absence of quality elements in the drug use prevention projects and each project’s approximate quality index.

**Table 3:** Present and absent quality elements in drug use prevention projects in Vitória (ES) schools

Project		1	2	3	4	5	6	7	8	9	10	11	12	13	14	15	16	% Yes	% No
Quality element																			
Theoretical foundation	1. Theoretical model	Y	N	Y	N	Y	Y	Y	Y	N	Y	Y	Y	Y	Y	Y	Y	81.25	18.75
Objectives	2. Clear definition	Y	Y	Y	Y	Y	Y	Y	Y	Y	Y	Y	Y	Y	Y	Y	Y	100	0
	3. Focus on primary prevention	N	N	N	N	N	Y	N	N	N	N	N	N	Y	Y	N	N	18.75	81.25
	4. Focus on secondary prevention	Y	Y	Y	Y	Y	Y	Y	Y	N	Y	Y	Y	Y	Y	Y	Y	93.75	6.25
	5. Focus on tertiary prevention	N	N	N	N	N	N	N	N	N	N	N	N	N	N	N	N	0	100
Participants	6. Families	N	N	N	Y	N	Y	Y	N	N	N	N	Y	Y	Y	N	N	37.5	62.5
	7. Out-of-school community	N	N	N	N	N	N	N	N	N	N	N	N	N	N	N	N	0	100
	8. Elementary school students	N	Y	N	Y	Y	Y	N	N	N	Y	N	Y	Y	Y	N	Y	56.25	43.75
	9. Secondary school students	Y	N	N	Y	Y	Y	Y	N	N	N	Y	N	N	N	Y	N	43.75	56.25
Content	10. Life skills	N	N	N	N	N	Y	N	N	N	N	N	N	Y	N	N	N	12.5	87.5
	11. Knowledge about drugs	Y	Y	N	N	Y	Y	Y	Y	Y	Y	Y	Y	Y	Y	Y	Y	87.5	12.5
	12. Positive social relationships	Y	N	Y	Y	N	Y	N	N	N	N	N	Y	Y	Y	N	N	43.75	56.25
	13. Alternative activities to drug use	N	N	Y	N	N	Y	Y	N	N	N	Y	Y	Y	Y	N	N	43.75	56.25
Methodology	14. Interactive/experiential	Y	Y	Y	Y	Y	Y	Y	Y	N	Y	Y	Y	Y	Y	Y	Y	93.75	6.25
	15. Informative	Y	Y	Y	Y	Y	Y	Y	Y	N	Y	Y	Y	Y	Y	Y	Y	93.75	6.25
	16. Written printed material	Y	N	Y	N	Y	Y	Y	Y	N	Y	N	N	Y	Y	Y	N	62.5	37.5
	17. Audio-visual/internet material	Y	N	Y	N	Y	Y	Y	N	N	N	N	N	Y	Y	Y	N	50	50
Characteristics	18. Bimonthly duration	N	N	Y	N	N	N	N	N	N	Y	Y	N	N	N	N	N	18.75	81.25
	19. Semi-annual duration	N	N	N	N	Y	N	N	Y	N	N	N	Y	Y	N	Y	N	31.25	68.75
	20. Annual duration	Y	N	N	N	N	Y	N	N	N	N	N	N	N	Y	N	N	18.75	81.25
	21. Daily periodicity	N	N	N	N	N	N	N	N	N	N	N	N	N	N	N	N	0	100
	22. Weekly periodicity	Y	N	N	Y	N	N	N	N	N	N	N	N	Y	N	N	N	18.75	81.25
	23. Monthly periodicity	Y	N	N	N	N	Y	N	Y	N	Y	Y	Y	N	Y	N	N	43.75	56.25
	24. Semi-annual periodicity	N	N	N	N	N	N	N	N	N	N	N	N	N	N	N	N	0	100
	25. Implementation by agents with specific training	Y	N	Y	N	N	N	Y	Y	N	Y	Y	Y	Y	Y	Y	N	62.25	37.75
	26. Implementation by out-of-school agents	Y	N	Y	N	N	N	Y	Y	N	Y	Y	Y	Y	Y	Y	N	62.25	37.75
	27. Implementation by students as mediators	Y	N	N	Y	N	N	N	N	N	Y	N	Y	N	Y	Y	N	37.5	62.5
	28. Design by the whole school community	N	N	N	N	N	N	N	N	N	N	N	N	N	Y	Y	N	12.5	87.5
	29. Design based on the participants’ needs	Y	N	Y	Y	Y	N	Y	Y	N	Y	Y	N	Y	Y	Y	Y	75	25
	30. Inclusion in the school curriculum	N	N	N	N	Y	N	Y	N	N	Y	Y	N	Y	N	Y	N	37.5	62.5
	31. Content reinforcement	N	N	N	N	N	N	N	N	N	N	N	N	N	N	N	N	0	100
Evaluation	32. Pre- and post-test	N	N	N	N	N	N	N	N	N	N	N	N	N	N	N	N	0	100
	33. Process evaluation	N	N	N	N	N	N	Y	N	N	N	Y	N	N	Y	Y	Y	31.25	68.75
	34. Control group	N	N	N	N	N	N	N	N	N	N	N	N	N	N	N	N	0	100
	35. Qualitative evaluation	N	N	N	N	N	N	Y	N	N	N	Y	N	N	Y	Y	Y	31.25	68.75
Total among 19 effectiveness elements: 1, 6, 10 to 17, 22 to 24 [considered one and any “intensity”], 27 to 33, 34 to 37 [considered one and any “evaluation”]		12	3	10	7	7	11	13	9	1	11	11	11	15	16	13	5		
Approximate quality index of the project		0.63	0.16	0.53	0.37	0.37	0.58	0.68	0.47	0.05	0.58	0.58	0.58	0.79	0.84	0.68	0.26		

Table 3 reports that the theoretical foundation was present in most projects (81.25%). Regarding the objectives, 100% of the projects had clearly defined objectives, and secondary prevention was the focus in 93.75% of them. The target population of the intervention coincided with the education level offered by most of the schools: elementary school students (56.25%). The family was not defined as the target of the intervention in 62.5% of the projects.

In regard to the content, emphasis was given to knowledge about drugs (87.5%); other contents related to the effectiveness of drug use prevention were absent from the projects, including life skills (absent in 87.5% of the projects) and positive social relationships and alternative activities to drug use (absent in 56.25% of the projects). Methodologically, most projects (93.75%) proposed interactive/experiential educational activities; printed materials were used in 62.5% of the projects, and audio-visual resources/internet in half of them.

Most interventions (31.25%) were planned to take place during one semester, though the specification of the intervention duration is absent in five projects; monthly was the most adopted periodicity (43.75%). The implementation time was indicated in only one project. The majority (75%) had the effectiveness element “design based on the participants’ needs”; the majority (62.25%) also indicated the following effectiveness elements of school-based prevention: “interventions planned to be performed by agents with specific training” and/or “by out-of-school agents”. However, the effectiveness element “participation of students as mediators” was absent in most projects (62.5%). Almost all of the projects (87.5%) did not have the effectiveness element “designed by the entire school community”. Other absent effectiveness elements were included in 62.5% of the projects, the planned activities were not included in the school curriculum, and content reinforcement was not included in any of the projects. Qualitative process evaluation was present in a few projects (31.25%).

Among the 16 projects, 10 achieved an approximate quality index above 0.50. The approximate quality index of the project enabled ranking the projects by quality, with project identified by number 14, “*Viva mais: as drogas dão asas, mas não ensinam a voar*” (“Live more: drugs give wings, but they do not teach how to fly”), displaying the highest quality index (0.84).

## Discussion

This study analysed the quality of school-based drug use prevention projects in Vitória, state of Espírito Santo (ES), Brazil, to propose improvements in those projects, according to the absence of strength-of-evidence of effectiveness elements (Jiménez et al. 2014). This analysis is discussed in its general aspects and its aspects that need improvements.

In general, projects explain one prevention theory, namely health education, which is among those theories most related to effectiveness (Espada et al. 2015). This element is related to effectiveness, regardless of the theory (Ramos et al. 2010) because as a rule, it will guide decision-making in project implementation (Winters et al. 2007).

Secondary prevention is the focus in almost all projects. A large part of the target schools is in drug trafficking areas; therefore, students are more vulnerable to the risk factors (Fabrizio et al. 2013; MacLean et al. 2013). In the introductions of the projects, in general, that fact is stated along with the profile of the students (e.g. level of engagement with school) and the difficulty of family participation in school. This finding indicates the presence of the effectiveness element “design based on the participants’ needs” (Jiménez et al. 2014); however, more than 60% of the projects do not include the family as a target of the intervention. The family should be actively involved in the project, at least in some of the proposed activities, given its impact in reducing the likelihood of inappropriate behaviour by the students (Peters et al. 2009).

Knowledge about drugs is included as a content in most projects, and the informative methodology used for participants to access the knowledge is always interactive/experiential, a quality indicator (Espada et al. 2015.), regardless of the methodological variability (cf. Griffin and Botvin 2010; Jackson et al. 2012.). The use of printed and audio-visual materials is quite frequent and improves the quality of most projects due to the relationship between these elements and effectiveness (Espada et al. 2015).

However, the content adopted must be more carefully considered regarding the quality. The primary element of content quality (“life skills”, Winters et al. 2007) is absent in most projects. The same goes for “positive social relationships” and “alternative activities to drug use”. The former are contents of actions aimed at strengthening the positive social student-student, students-teachers, students-parents and teachers-parents networks (Jiménez et al. 2014), which function as inhibitors of the likelihood of drug use by the students (the positive school ethos, Markham et al. 2012).

The formal characteristics of the projects are also worthy of attention. The effectiveness element intensity was difficult to identify, as already noted by Gottfredson and Wilson (2003). In this study, intensity was inferred from the frequency and periodicity of the activities (usually monthly). These temporal aspects are relevant because preventive interventions should be continuous, given that their effects tend to be diluted or disappear (Gázquez et al. 2009). This feature is related to the other absences: content reinforcement (100%) and inclusion in the curriculum (62.5%). There seems to be a disconnection between the prevention program and the pedagogical project, which explains the non-involvement of the entire school community in the design of 87.5% of the projects. These data go against the main theoretical model that underlies most projects because “the participation of all school community members is an essential principle that underpins health promotion practice” (Rowling 2003, p. 17).

A positive aspect is that the activities are expected to be implemented by agents with specific training and/or by out-of-school agents, showing the partnership between school and community, which increases the likelihood of an effective intervention (Sandler et al. 2014). A negative aspect is that students are excluded as mediators in 62.5% of the projects. Although this effectiveness element is controversial (Jiménez et al. 2014), effective interventions had the participation of the students (Fernández et al. 2002); in fact, mediation made only by students was more effective (Gottfredson and Wilson 2003).

Finally, the evaluation process is poorly described, i.e. the measurement of what would be an expected result is absent (Winters et al. 2007). In general, needs deriving from a realistic prevention plan are described, but changes to those needs are never considered for evaluating the fulfilment of this plan. Only five projects predict a qualitative process evaluation.

Data analysis showed that life skills content, participation of students as mediators, positive social relationships content, participation of families, quality evaluation, content reinforcement and design by the whole school community are the elements of effectiveness less present in the projects. The projects in which these elements are absent need improvement, in particular in “life skills content” and “design by the whole school community” elements, absent in 87.5% of the projects.

Life skills content cover personal and social and, directly associated with drug resistance, health-related competences. Those competences (e.g. communication, decision-making, coping and stress management are the element of effectiveness with the more weight of the evidence, Jiménez et al. 2014). They are also involved with other elements of effectiveness such as to positive social relationships content, alternatives to drug use and participation of families. Improvements in this content must consider principles of best practice in health education, experiential learning and flexibility to cultural-family aspects (Ramos et al. 2010). The active inclusion of the family in prevention activities is justified by the effectiveness of its inclusion (Peters et al. 2009). Some projects already have some concrete measures in this regard, as suggested by Ramos et al. (2010), including (a) presenting the project to the parents, suggesting their participation, including changing and preparing the activities; (b) feedback on, for example, survey results or students’ performance; and (c) awareness of their roles as models of positive behaviour regarding drug use.

Content shortcomings indicate the need to include objectives for improving the positive social networks of students. Due to the preventive benefits of family interactions, the strengthening of the positive family relationships should be the “primary purpose” of drug use prevention programs (Ramos et al. 2010, p. 94). The primacy of this fact is related to the centrality of life skills in prevention (Winters et al. 2007). Concrete measures for the improvement of this shortcoming, with the participation of parents whenever possible, include (a) sporting, entertainment and leisure activities; (b) volunteer activities in social welfare services (in any place where one can be supportive); and (c) social skills training activities (cf. Del Prette and Del Prette 2011).

“Students as mediators” is a quality element of prevention projects (Fernández et al. 2002). Those students who do not use drugs feel conflicted when facing contingencies that favour self-use (Ramos et al. 2010). Moreover, by including adolescents in interventions targeting themselves, the program benefits from the well-publicized phenomenon of group identification (Viner et al. 2012). Ramos et al. suggest the inclusion of moments, as concrete measures in this context, in which the students, for example, (a) talk about their experiences; (b) are trained as mediators; and/or (c) coordinate activities.

The fact that only two projects (12.5%) have been designed by the whole school community is a serious shortcoming because good health promotion projects consist of actions that are continuously determined and implemented by everyone involved (Rowling 2003). The concrete measures that Ramos et al. suggested for this element include activities (a) of awareness of the importance of everyone—families, teachers, students, officers, and directors—to participate in the design and/or modification of the project and (b) involving the effective participation of everyone in the prevention process.

Gázquez et al. (2009) warn that the preventive effects of drug use typically do not last or gradually decrease over time. Therefore, reinforcement sessions should be planned in the project and implemented after the end of the intervention to achieve medium- and long-term effects. To achieve these effects, Ramos et al. suggest including at least three content reinforcement sessions in the project.

The absence of the element “evaluation” is another serious shortcoming of the projects. Evaluation in effective prevention projects is rigorous and an indicator of quality (Jiménez et al. 2014). According to Winters et al. (2007), the evaluation of risk factors precedes the definition of objectives of effective prevention, which include modifying changeable factors. Most analysed projects evaluate needs and include the evaluations in the intervention plan. However, the projects disregard whether the evaluated needs must be changed based on the intervention plan. Concrete measures to improve this shortcoming include defining two phases in the project for obtaining outcome measures (e.g. “I now” versus “I then” essays) or process measures (e.g. observation of the participants’ behaviour).

## Conclusions

More than half of the drug use prevention projects (62.5%) in Vitória, ES, schools reached an approximate quality index above 0.50. The main quality indicators are the presence of (a) a theoretical foundation (health education model); (b) interactive/experiential methodologies; (c) design based on the participants’ needs; and (d) implementation by agents with specific training and/or out-of-school agents. The main indicators of shortcomings are the absence of (a) families as participants; (b) life skills, positive relationships and alternative activities to drug use as content; (c) implementation by students as mediators; (d) design by the whole school community; (e) inclusion in the school curriculum; (f) content reinforcement; and (g) evaluation. This article presented improvement proposals with concrete measures to prevent these shortcomings. Despite that more than half of the drug use prevention projects have reached a good quality index, the absent indicators of shortcomings must be included in them or in new projects. The weight of the evidence of these indicators allows creating a hierarchy of elements to improve the quality of school-based preventive interventions: life skills content, implementation by students as mediators, families as participants and positive relationships content, content reinforcement and design by the whole school community (Jiménez et al. 2014).

In turn, the shortcomings of this study are related to the non-inclusion of elements from the teacher training process, which could have provided data on motivational and knowledge factors for writing the projects. Further analysis of these factors and the consideration of this study’s findings in the implementation of such projects and the writing of new projects can possibly improve the continuing education policy for drug use prevention aimed at teachers in Brazil.
